# Antiretroviral drugs induce oxidative stress and neuronal damage in the central nervous system

**DOI:** 10.1007/s13365-013-0227-1

**Published:** 2014-01-14

**Authors:** Cagla Akay, Michael Cooper, Akinleye Odeleye, Brigid K. Jensen, Michael G. White, Fair Vassoler, Patrick J. Gannon, Joseph Mankowski, Jamie L. Dorsey, Alison M. Buch, Stephanie A. Cross, Denise R. Cook, Michelle-Marie Peña, Emily S. Andersen, Melpo Christofidou-Solomidou, Kathryn A. Lindl, M. Christine Zink, Janice Clements, R. Christopher Pierce, Dennis L. Kolson, Kelly L. Jordan-Sciutto

**Affiliations:** 1 Department of Pathology, School of Dental Medicine, University of Pennsylvania, 240 S. 40th St, Rm 312 Levy Bldg, Philadelphia, PA 19104-6030 USA; 2 Department of Psychiatry, The Perelman School of Medicine, University of Pennsylvania, Philadelphia, PA USA; 3 Department of Molecular and Comparative Pathobiology, Johns Hopkins University School of Medicine, Baltimore, MD USA; 4 Department of Neurology, The Perelman School of Medicine, University of Pennsylvania, Philadelphia, PA USA; 5 Department of Medicine, The Perelman School of Medicine, University of Pennsylvania, Philadelphia, PA USA

**Keywords:** Antiretroviral, Fumaric acid ester, HIV, HIV-associated neurocognitive disorder, Macaque, Oxidative Stress, Reactive oxygen species, SIV

## Abstract

HIV-associated neurocognitive disorder (HAND), characterized by a wide spectrum of behavioral, cognitive, and motor dysfunctions, continues to affect approximately 50 % of HIV(+) patients despite the success of combination antiretroviral drug therapy (cART) in the periphery. Of note, potential toxicity of antiretroviral drugs in the central nervous system (CNS) remains remarkably underexplored and may contribute to the persistence of HAND in the cART era. Previous studies have shown antiretrovirals (ARVs) to be neurotoxic in the peripheral nervous system in vivo and in peripheral neurons in vitro. Alterations in lipid and protein metabolism, mitochondrial damage, and oxidative stress all play a role in peripheral ARV neurotoxicity. We hypothesized that ARVs also induce cellular stresses in the CNS, ultimately leading to neuronal damage and contributing to the changing clinical and pathological picture seen in HIV-positive patients in the cART era. In this report, we show that ARVs are neurotoxic in the CNS in both pigtail macaques and rats in vivo. Furthermore, in vitro, ARVs lead to accumulation of reactive oxygen species (ROS), and ultimately induction of neuronal damage and death. Whereas ARVs alone caused some activation of the endogenous antioxidant response in vitro, augmentation of this response by a fumaric acid ester, monomethyl fumarate (MMF), blocked ARV-induced ROS generation, and neuronal damage/death. These findings implicate oxidative stress as a contributor to the underlying mechanisms of ARV-induced neurotoxicity and will provide an access point for adjunctive therapies to complement ARV therapy and reduce neurotoxicity in this patient population.

## Introduction

Despite introduction of combination antiretroviral therapy (cART), HIV-associated neurocognitive disorder (HAND) continues to affect approximately 50 % of HIV(+) patients (Dore et al. 1999; Heaton et al. 2010). Furthermore, in the cART era, the underlying neuropathology has shifted from overt subcortical involvement to a more insidious cortical damage (Gannon et al. 2011). Various factors, such as poor adherence to drug regimen, emergence of resistant virus species, and residual viral DNA in the central nervous system (CNS), may contribute to these changes (Gannon et al. 2011). However, another likely contributor to HAND in the cART era is the virtually unstudied potential for antiretroviral (ARV)-related toxicity in the CNS. cART has decreased HIV-related morbidity and mortality by limiting T cell loss and controlling opportunistic infections. However, cART regimens are associated with potentially serious side effects, including dyslipidemia, lipohypertrophy, and increased risk of atherosclerosis (Vidal et al. 2010). Additionally, cART-associated toxicity in the peripheral nervous system is well documented, and it is likely that cART would trigger similar responses in the CNS. Pharmacokinetic studies suggest limited ARV penetrance into the CNS and indicate low cerebrospinal fluid (CSF) and parenchymal drug concentrations (Yilmaz et al. 2004; Yilmaz et al. 2009). However, direct blood–brain barrier (BBB) compromise by viral proteins and neuroinflammation and indirect BBB impairment due to concomitant factors, such as coexisting infections, can lead to increased CSF and parenchymal drug concentrations. Thus, the impact of ARVs in the CNS of HIV(+) patients is clinically relevant and must be examined.

Initial cART usually includes two nucleoside/nucleotide reverse-transcriptase inhibitors (NRTIs) in combination with a non-nucleoside reverse-transcriptase inhibitor (nNRTI) or with a protease inhibitor (PI) boosted with a low dose of a second PI, Ritonavir. NRTIs and nNRTIs bind to the HIV reverse-transcriptase enzyme and inhibit proviral DNA synthesis; PIs inhibit viral proteases needed for virus maturation and assembly. Despite some crossover, certain ARV classes are more highly associated with particular side effects and toxicities than are other classes. PIs alter lipid metabolism and induce the endoplasmic reticulum stress response in macrophages, linking PIs to increased risk of atherosclerosis (Touzet and Philips 2010). NRTIs inhibit DNA polymerase γ and lead to decreased mitochondrial DNA, loss of mitochondrial membrane potential, and oxidative phosphorylation, consequently precipitating oxidative stress (Nolan and Mallal 2004). Previous studies exploring possible side effects of ARVs in the CNS are scarce and mostly involve cell lines (Cui et al. 1997). Given the mutations and aberrations in immortalized cell lines, these studies may not reflect the ARV toxicity potentially occurring in biological settings. Peripheral dorsal root ganglia neurons are the only primary cell type of neural lineage previously studied for ARV toxicity (Werth et al. 1994). In this report, we examined effects of ARVs in primary CNS neurons both in vivo and in vitro. Our findings suggest that cART induces oxidative stress and neurotoxicity in the CNS, and that the patients on long-term cART regimens would benefit from adjuvant therapies that include antioxidant strategies to overcome deleterious effects of cART in the CNS.

## Materials and methods

### Chemicals and reagents

Chemicals and reagents comprise the following: (1) AIDS Research and Reference Reagent Program, Division of AIDS, NIAID, NIH, antiretroviral reagents; (2) Abcam (Cambridge, MA), mouse monoclonal NAD(P)H/quinone oxidoreductase-1 (NQO-1) antibody (A180) and mouse monoclonal synaptophysin antibody (SY38); (3) BioRad (Hercules, CA), Biosafe Coomassie stain, immunoblot polyvinylidene fluoride (PVDF) membrane and prestained broad range molecular weight ladder; (4) Cell Signaling Technology (Danvers, MA), rabbit polyclonal antibody raised against cleaved caspase-3; (5) Citifluor, Ltd. (London, UK), citifluor AF1. Covance (Princeton, NJ), mouse monoclonal microtubule-associated protein 2 (MAP2) antibody (SMI-52); (6) Dako (Carpinteria, CA), rabbit polyclonal glial fibrillary acidic protein (GFAP) antibody (Z0334); (7) Enzo Life Sciences (Farmingdale, NY), rabbit polyclonal antibody to heme-oxygenase-1 (HO-1); (8) Frontier Scientific (Logan, UT), Sn(IV) mesophorphyrin IX dichloride (SnMP); (9) Jackson ImmunoResearch Labs (West Grove, PA), fluorescein isothiocyanate-conjugated goat anti-mouse IgG and Cy3-conjugated goat anti-rabbit IgG secondary antibodies; (10) Invitrogen (Carlsbad, CA), Dulbecco's modified Eagle's medium (DMEM), tetramethyl rhodamine methyl ester, goat anti-mouse beta-lactamase TEM-1 conjugate, fluorocillin green substrate, dihydroethidium (DHE), 4′,6-diamidino-2-phenylindole (DAPI), neurobasal media, and B27 supplement; (11) New England Biolabs (Ipswich, MA), tyramide amplification system; (12) Peptide International (Louisville, KY), poly-l-lysine; (13) Sigma Aldrich (St. Louis, MO), carbonyl cyanide *m*-chlorophenyl hydrazone, cytosine arabinoside (Ara-C), fetal bovine serum (FBS), monomethylfumarate (MMF), oligomycin, propidium iodide, and PI cocktail; (14) ScyTek Labs (Logan, UT), normal antibody diluent; (15) Thermo Scientific (Waltham, MA), goat anti-rabbit horse radish peroxidase (HRP) antibody and goat anti-mouse HRP antibody, SuperSignal West Dura extended duration substrate; and (16) Tocris Bioscience (Ellisville, MO), thapsigargin. The antibody against calpain-cleaved spectrin was a generous gift from Dr. Robert Siman (The Perelman School of Medicine, University of Pennsylvania, Philadelphia, PA).

### Primary cortical neuroglial cultures

Primary rat cortical neuroglial, pure neuronal, and pure astrocytic cultures were isolated from embryonic day 17 Sprague Dawley rat pups, with modifications of protocols previously described (Wilcox et al. 1994). Briefly, the cortical cell suspensions isolated from rat pups following standard protocols were plated on poly-l-lysine-coated T25 tissue culture flasks (2 × 10^6^ cells/flask), 96-well tissue culture plates (0.5 × 10^5^ cells/well), or glass coverslips (0.25 × 10^6^ cells/well). These cultures were maintained in neurobasal media with B27 supplement at 37 °C with 5 % CO_2_ for the generation of neuroglial cultures, as described previously (Wang et al. 2010; Akay et al. 2011b; White et al. 2011). Half of the media was replaced with fresh media every 7 days and the experiments were performed at 21 days in vitro (DIV), at which time the cultures contain approximately 85–90 % neurons and 10–15 % astrocytes/glia. For the preparation of pure neuronal cultures, cultures were treated with 10 μM Ara-C 48 h after plating and maintained in neurobasal media with B27 supplement at 37 °C with 5 % CO_2_; at 21 DIV, the age at which the experiments were conducted, no astrocytes are detectable by staining for GFAP. Pure astrocytic cultures were prepared by initially plating the cortical cell suspensions in 10 % FBS in DMEM and maintaining at 37 °C with 5 % CO_2_ for 7–12 days, after which time astrocytes constitute more than 90 % of the cultures. These pure astrocytic cultures are then replated in T25 flask or poly-l-lysine-coated glass coverslips in DMEM/10 % FBS and the experiments are conducted 3–5 days after replating.

### SIV/pigtail macaque model of HIV CNS disease

Juvenile pig-tailed macaques (*Macaca nemestrina*) were inoculated with SIV/DeltaB670 and SIV/17E-Fr, as described previously (Zink et al. 1999). Beginning 12 days after inoculation, animals were treated daily with a four ARV drug combination (cART) until necropsy (range, days 161–175). The treatment consisted of the NRTI tenofovir (Gilead) at 30 mg/kg subcutaneously every day; the PIs saquinavir (Roche) and atazanavir (Bristol- Myers Squibb) at 205 and 270 mg/kg orally twice a day, respectively; and the integrase inhibitor L-870812 (Merck)(Hazuda et al. 2004) at a dose of 10 mg/kg orally twice a day(Zink et al. 2010). The tenofovir dose was determined on the basis of previous studies(Tsai et al. 1997), whereas atazanavir and saquinavir doses were determined by pharmacokinetic experiments conducted in pig-tailed macaques, and reflected those that resulted in the same area under the curve as detected in humans treated with atazanavir and saquinavir (Zink et al. 2010). The dose of the integrase inhibitor was based on a previous study conducted in rhesus macaques(Hazuda et al. 2004).

### Rodent model of antiretroviral-induced neurotoxicity

All surgical procedures were performed with the approval of the Institutional Animal Care and Use Committee. Adult male Sprague Dawley rats were catheterized via jugular vein, as described previously (Thrivikraman et al. 2002). The drug cocktail composed of AZT (100 mg kg^−1^ day^−1^), ritonavir (20 mg kg^−1^ day^−1^), and saquinavir (25 mg kg^−1^ day^−1^) was administered twice daily for 7 days by continuous intravenous injection. Each drug dose was based on previously published studies in rodents (Shibata et al. 2002; Huisman et al. 2003; Manda et al. 2010; Pistell et al. 2010; Waring et al. 2010; Yang et al. 2010; du Plooy et al. 2011; Fontes et al. 2011; Lledo-Garcia et al. 2011; Wagner et al. 2011; Mak et al. 2013; Reyskens and Essop 2013a, b; Reyskens et al. 2013). The catheters were flushed with 0.3 ml heparin (50 IU/ml) in PBS until the end of treatments. At time of euthanasia, preceding decapitation and tissue harvest, catheter patency was reverified by response to pre-euthanasia sedation (100 mg/kg ketamine per 10 mg/kg xylazine). The brains were removed, the frontal cortex and the hippocampus were dissected on ice, and the tissue samples were stored at -80 °C until immunoblot analysis.

### Immunofluorescence staining of tissue

Tissue slides of paraffin-embedded tissue sections from hippocampus of male and female pig-tailed macaques (*M. nemestrina*) were prepared for immunofluorescent staining, as described previously (Akay et al. 2011a) . Briefly, glass slides containing paraffin-embedded sections (10 μm) were heated to 55 °C for at least 30 min, deparaffinized in Histoclear, and rehydrated with consecutive 100, 95, 90, and 70 % ethanol washes. Endogenous peroxidase activity was blocked with 3 % H_2_O_2_ in methanol and antigen unmasking was achieved with target retrieval solution at 95 °C for 1 h. Tissue sections were blocked with 10 % normal goat serum in phosphate-buffered saline solution (PBS). Mouse monoclonal antibodies to synaptophysin, MAP2, and rabbit polyclonal antibody to GFAP were used at empirically defined dilutions (synaptophysin at 1:500; MAP2 at 1:100; and GFAP at 1:80), and DAPI was used to stain nuclei. The tyramide amplification system was used to detect synaptophysin. Slides were mounted in Citifluor AF1, and for each specimen, five to ten randomly selected areas within the CA1–CA3 layer of the hippocampus were scanned along the *z*-axis to create z-stack images at high magnification (×600) by laser confocal microscopy on a Biorad Radiance 2100 equipped with Argon, Green He/Ne, Red Diode, and Blue Diode lasers (Biorad, Hercules, CA). Post-acquisition analysis was conducted using MetaMorph 6.0 (Universal Imaging, Downingtown, PA). Total intensity for synaptophysin, MAP2 and GFAP were determined by the measurement of integrated pixel intensity for synaptophysin, MAP2 or GFAP per *z*-stack image, where the integrated pixel intensity is defined as total pixel intensity per image times the area of pixels positive for synaptophysin, MAP2 or GFAP. Averages are expressed as mean ± SEM. All data was analyzed by Prism 5.0 software (GraphPad Software, San Diego, CA).

### MAP2 cell-based ELISA

A MAP2 cell-based ELISA was performed to quantify neuronal damage/death as previously described (White et al. 2011). Briefly, primary rat cortical neuroglial cultures plated in 96-well plates were fixed for 30 min with 4 % paraformaldehyde in 4 % sucrose at the conclusion of treatments. After blocking for 1 h with 5 % normal goat serum in PBS, the plates were incubated with monoclonal MAP2 antibody overnight at 4 °C, followed by washes with PBS with 0.1 % Tween-20 (PBS-T). The plates were then incubated for 30 min with goat anti-mouse secondary antibody conjugated to beta-lactamase TEM-1 at room temperature (RT), washed with PBS-T, and incubated in the dark at RT for 1 h in fluorocillin green substrate. Fluorescence intensity was measured using a Fluoroskan Ascent fluorometer plate reader (Thermo Electron, Waltham, MA) with excitation at 485 nm and emission at 527 nm.

### Hand counting of MAP2-positive cells

As a complementary method to MAP2 ELISA, neuronal survival was verified by hand counting of MAP2-positive neurons; 15 μM propidium iodide was added to primary cortical neuroglial cultures grown on coverslips 15 min before the end of the treatments. The coverslips were washed once with PBS and fixed for 30 min with 4 % paraformaldehyde in 4 % sucrose, followed by blocking and permeabilization in 0.2 % BSA + 0.1 % Triton X-100 in PBS for 1 h at RT. The coverslips were then washed twice with PBS and incubated with anti-MAP2 antibody (1:100) in normal antibody diluent for 2 h at RT. After two washes with PBS-T, the coverslips were incubated in a fluorescein isothiocyanate-conjugated goat anti-mouse IgG secondary antibody (1:200) for 30 min at RT. The coverslips were mounted on slides and alive/dead neurons were hand-counted based on MAP2 and PI-positive staining using a Nikon Eclipse E400 fluorescent microscope (Nikon Corp, Tokyo, Japan) equipped with Olympus DP70 digital camera (Olympus Corp, Tokyo, Japan). Live cells stain negative for PI, while dead cells retain it in their nuclei. The number of surviving MAP2-positive neurons will be positive for MAP2 and negative for PI staining. The percentage of MAP2-positive cells ± SEM were calculated from blinded counting of six fields at ×200 in two adjacent vertical columns through the center of each coverslip, proceeding from top to bottom and bottom to top. For each condition, three coverslips were counted from two or more independent experiments.

### Quantification of synaptophysin

Cultures grown on coverslips, as described above, were immunofluorescently stained for synaptophysin (1:1,000), MAP2 (1:100), and DAPI. Tyramide amplification was used for the detection of synaptophysin. For each treatment condition, images of 10 randomly selected areas from three coverslips from three independent experiments were captured by fluorescence microscopy. Post-acquisition analysis was performed using NIH ImageJ program (V 1.36b, Bethesda, MD). Briefly, synaptophysin-positive puncta were detected by background subtraction and manual thresholding. The number of synaptophysin-positive puncta was determined by dividing the total number of puncta within a dendrite segment by the length of the segment.

### Measurement of reactive oxygen species

The superoxide indicator dihydroethidium (DHE, Invitrogen) was used to detect the presence of reactive oxygen species (ROS) in vitro. 3 μM DHE was added to culture media 15 min prior to conclusion of treatments. Cells were then washed with PBS, fixed with 4 % paraformaldehyde in 4 % sucrose at RT for 8 min, and stained for DAPI. The coverslips were then mounted on slides with CytoSeal and visualized using fluorescent microscopy, as described above. Post-acquisition analysis was performed using MetaMorph to determine the fluorescence intensity of DHE normalized to the area of DAPI signal.

### Immunoblotting

Whole cell extracts of rat tissue samples were prepared by homogenization in ice-cold tissue extraction buffer (50 mM Tris at pH 7.5, 0.5 M NaCl, 1 % NP-40, 1 % SDS, 2 mM EDTA, 2 mM EGTA, 5 mM NaF, 0.4 mM Na_3_VO_4_, 1 mM dithiothreitol (DTT), and 1:100 PI cocktail), followed by centrifugation at 12,000×*g* at 4 °C for 20 min. Whole cell extracts of primary rat cortical cultures were prepared with ice-cold cell lysis buffer (50 mM Tris at pH 7.5, 120 mM NaCl, 0.5 % NP-40, 10 mM EDTA, 0.4 mM Na_3_VO_4_, 100 mM DTT, and 1:100 PI cocktail), followed by centrifugation at 14,000×*g* at 4 °C for 10 min. The protein concentrations of the collected supernatants were determined using the Bradford method and 25–50 μg of protein was loaded into each lane of a 4–12 % Bis-Tris gradient gel for separation. A broad range molecular weight ladder was run on each gel. Subsequent to separation, proteins were transferred onto PVDF membranes, and blocked in Tris-buffered saline with 0.1 % Tween-20 (TBS-T) and 5 % bovine serum albumin (BSA) for 1 h at RT. The membranes were incubated with the primary antibodies in TBS-T with 5 % BSA at 4 °C overnight, washed with TBS-T, followed by incubation with corresponding HRP-conjugated secondary antibodies. The membranes were developed using SuperSignal West Dura extended duration substrate. Loading controls were obtained by staining the membranes and the gels with the Biosafe Coomassie Stain for 20 min, followed by destaining with deionized water for 30 min. For densitometric analysis, autographs were scanned into Adobe Photoshop (Adobe Systems, San Jose, CA) and regions of interests (ROI) of equal size were determined for each band. The pixel intensities of ROIs were quantified using the NIH ImageJ program (V 1.36b, Bethesda, MD). Target band intensities were normalized to gel and membrane coomassie stain controls to account for protein degradation and loading discrepancies, and the normalized target band intensities were used to quantify fold changes over controls.

### Quantitative reverse transcription polymerase chain reaction

The expression of HO-1 and NQO-1 genes in rat neuroglial cells was quantified by quantitative reverse transcription polymerase chain reaction (qRT-PCR). Custom TaqMan® Gene Expression Assays were purchased from Applied Biosystems for the genes: NQO-1 (Rn00566528_m1) and HO-1 (Rn01536933_m1). Approximately 5 ng cDNA was used per reaction. StepOne™ Software v2.0 was used to construct the experimental protocol and the qRT-PCR took place in the StepOne Real-Time PCR System (Applied Biosystems, Carlsbad, CA). Data was normalized using both β-actin (Rn00667869_m1) and 18S (Hs99999901_s1) and was analyzed according to the ΔΔC_T_ method. All samples were run in triplicate from three biological replicates.

### Statistical analysis

All data was analyzed by Prism 5.0 software (GraphPad Software, San Diego, CA). Values are expressed as mean ± SEM, and values of *p* < 0.05 were considered significant for all statistical analyses performed.

## Results

### Antiretroviral drugs lead to neuronal damage in vivo

**Fig. 1:**
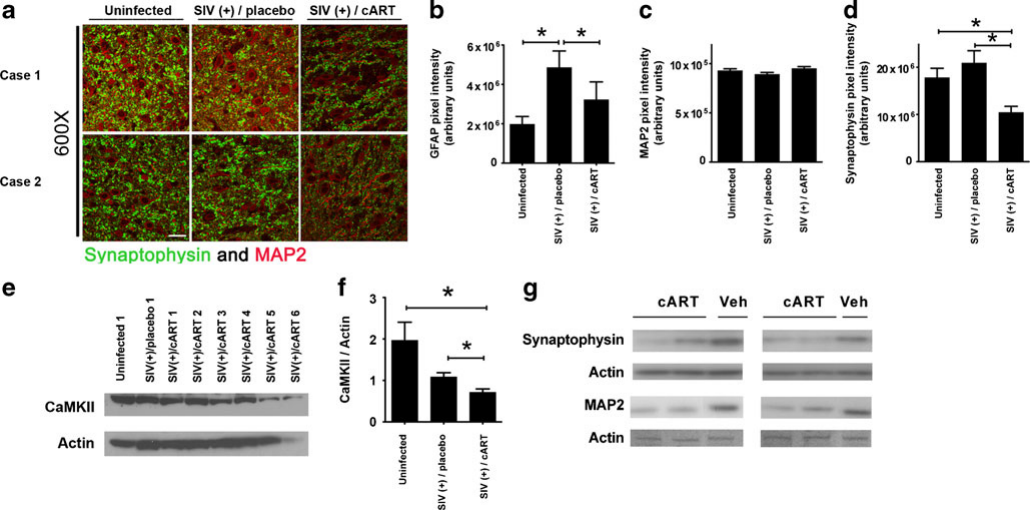
Antiretroviral drugs induce neuronal damage in vivo. **a**–**d** Formalin-fixed, paraffin-embedded tissue sections from hippocampus of pig-tailed macaques that were either uninfected (*n* = 6), SIV infected but not cART treated (*n* = 7), or SIV infected and treated with cART (tenofovir, atazanavir, saquinavir, and L-870812a; *n* = 4) were prepared for immunofluorescent analysis and were triple labeled for MAP2 (*red*), synaptophysin (*green*), and GFAP. Sections were visualized by laser confocal microscopy and images were quantified for MAP2, synaptophysin and GFAP expression. **a** Representative composite images of two cases per group which were stained with MAP2 and synaptophysin are shown. *Scale bar* = 30 μm. **b** Quantification shows the resolution of GFAP immunoreactivity in SIV(+)/cART group, compared with SIV(+)/placebo group (one-way ANOVA, *p < 0.05). No changes were observed in MAP2 expression between groups (**c**), but there were statistically significant decreases in synaptophysin immunoreactivity (**d**) in SIV(+)/cART group, as compared with SIV(+)/untreated and uninfected groups (one-way ANOVA, *p < 0.05, *ns* not significant). **e**, **f** Fresh-frozen tissue sections from the frontal cortex of pig-tailed macaques that were either uninfected (*n* = 3), SIV infected but not cART treated (*n* = 6), or SIV infected and cART treated (*n* = 6) were used for standard protein extraction and subsequent immunoblotting for the expression of CaMKII. Actin was used as a loading control. A representative immunoblot is shown. Quantification shows statistically significant decreases in CaMKII in the cART-treated group, as compared with the uninfected group or the SIV(+)/untreated group (one-way ANOVA, *p < 0.05). **g** Whole cell lysates prepared from hippocampus of rats treated for 7 days with AZT/Rit/Saq (*n* = 4) or vehicle (*n* = 2) were immunoblotted for synaptophysin and MAP2. A band from the coomassie blue staining is included to control for equal loading and protein degradation

cART-induced peripheral neuropathy is well documented (Power et al. 2009), and it is likely that cART triggers similar damage to neurons in the CNS. To determine the neurotoxic potential of cART in the CNS, we assessed the effects of an ARV regimen on the expression of synaptophysin and MAP2, indicators of synaptic damage and neuronal loss, respectively, utilizing post-mortem tissue from a well-characterized SIV/pigtail macaque model of HIV CNS disease, which was designed to address the efficacy of CNS penetrant antiretroviral therapy in reducing viral load in the CNS (Zink et al. 2010). In this study, animals infected with SIV either received no cART or received early cART treatment that included tenofovir (NRTI), atazanavir (PI), saquinavir (PI), and L-870812a (integrase inhibitor) 12 days after the virus inoculation. Without cART, 90 % of animals develop neurologic disease within 3 months postinoculation (p.i.). By contrast, animals receiving cART do not develop SIV encephalitis. Rather, they show a rapid reduction in their plasma and CSF viral load followed by continued suppression of SIV replication with maintenance of CD4+ T cell counts until elective euthanasia around day 160 p.i. Additionally, cART-treated animals do not exhibit any outward signs of neurological deficits. Further, our quantitative immunofluorescent analysis of hippocampal tissue sections revealed reduced astrogliosis in the hippocampus of SIV-infected, cART-treated animals (SIV(+)/cART), compared with SIV-infected macaques that did not receive cART ((SIV(+)/placebo)) (Fig. 1b). However, we observed statistically significant decreases in synaptophysin expression in the hippocampi of the SIV(+)/cART group, compared with that in either the uninfected or the SIV(+)/placebo group (Fig. 1a, d). In addition, examination of the expression of a second marker of synaptodendritic integrity, calmodulin kinase II (CaMKII), by immunoblotting showed that CaMKII levels were significantly lower in the frontal cortex in the SIV(+)/cART macaques than in their SIV(+)/placebo counterparts (Fig. 1e, f). CaMKII is highly expressed in neurons of macaque hippocampus and frontal cortex, whereas its expression in other cell types, including microglia, infiltrating macrophages, and multinucleated giant cells, is minimal; thus, the differences in CaMKII expression in frontal cortex of the animals from our experimental groups are neuron specific (Gupta et al. 2010). Our results demonstrate synaptic injury in the presence of cART despite effective control of SIV replication in the periphery and CNS. Interestingly, we did not observe changes in MAP2 fluorescence in the hippocampus of infected and/or cART-treated animals compared with untreated/uninfected animals (Fig. 1a, c).

As studies of uninfected, cART-treated macaques have not been performed to determine the contribution of cART to neuronal damage independent of SIV infection, we administered combinations of ARVs intravenously to adult rats. In patients, initial cART usually includes two nucleoside/NRTIs in combination with a nNRTI or with a PI boosted with a low dose of a second PI, ritonavir (Rit). Thus, we used zidovudine (AZT), an NRTI, along with two PIs, saquinavir (Saq) and Rit, at doses based on previously published pharmacokinetic studies of these ARVs (Busidan and Dow-Edwards 1999; Kageyama et al. 2005; Pistell et al. 2010). In agreement with previous studies (Waring et al. 2010), the animals showed no overt signs of distress during the course of the treatment. However, via immunoblotting, we observed decreases in hippocampal synaptophysin expression in cART-treated rats compared with vehicle-treated rats (Fig. 1g), complementing our findings of synaptic damage in SIV(+)/cART macaques and further supporting a role for ARV-associated neuronal injury in the CNS. Of note, we also detected decreases in MAP2 levels in the cART-treated rat hippocampus (Fig. 1g), which likely reflects the acute drug toxicity in this treatment paradigm, compared to the subtler synaptic injury observed in the cART-treated macaque brain.

### Antiretroviral compounds in therapeutically relevant combinations are neurotoxic in vitro

**Fig. 2:**
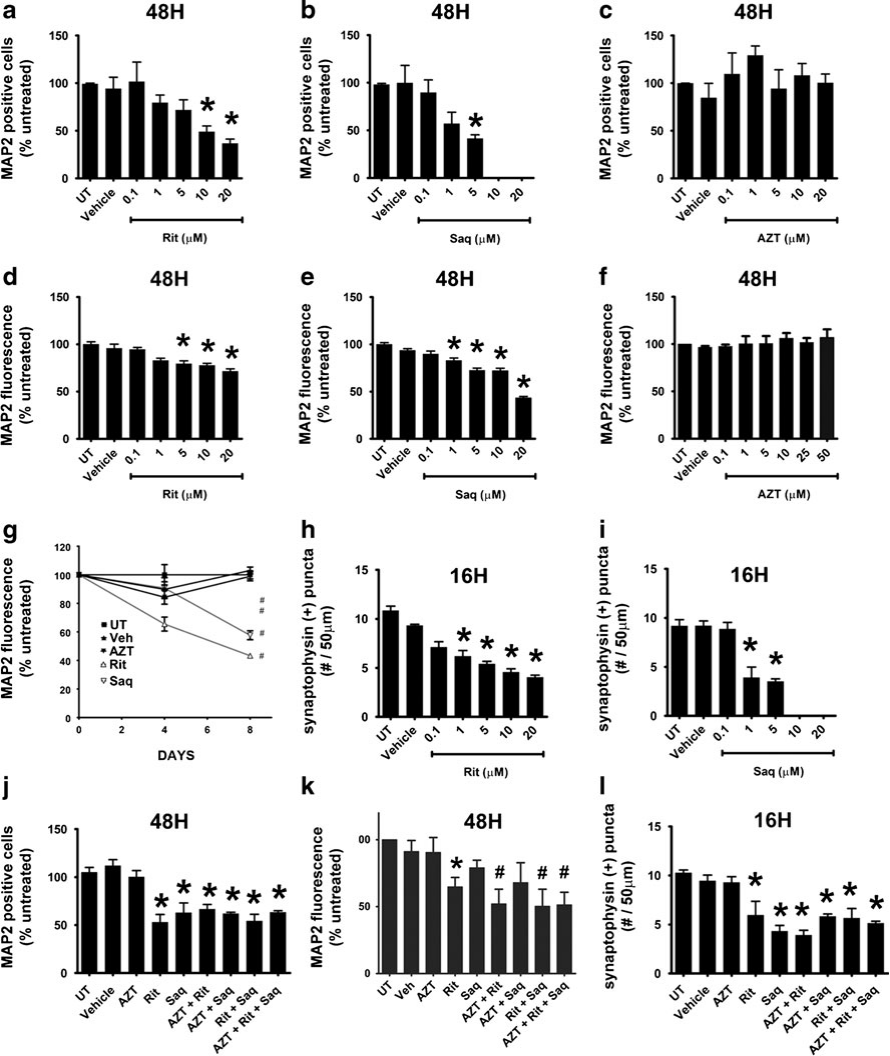
Therapeutically relevant combination antiretroviral drug treatments are neurotoxic in vitro. **a**–**c** Primary rat cortical neuroglial cultures aged 21 days in vitro (*DIV*) on coverslips were exposed to Rit (**a**), Saq (**b**), or AZT (**c**) at increasing doses for 48 h, followed by hand counting for MAP2-positive cells (*n* = 3; vehicle, 0.04 % DMSO; * *p* < 0.05, one-way ANOVA with post-hoc Newman–Keuls). **d**–**f** 21DIV primary neuroglial cultures grown in 96-well plates were treated with increasing doses of Rit (**d**), Saq (**e**), or AZT (**f**) for 48 h, followed by MAP2 cell-based ELISA (*n* = 2; vehicle, 0.04 % DMSO; * *p* < 0.05, one-way ANOVA with post-hoc Newman–Keuls). **g** Primary neuroglial cultures were treated with AZT (25 μM), Rit (10 μM), or Saq (1 μM) at day zero. Ninety percent of the media was changed with conditioned media supplemented with a fresh drug stock every 2 days, and cultures were analyzed by MAP2 cell-based ELISA at days 4 and 8 (*n* = 2; vehicle, 0.04 % DMSO; * *p* < 0.05, one-way ANOVA with post-hoc Newman–Keuls). **h**, **i** Cultures grown on coverslips were exposed to the indicated treatments and synaptophysin-positive puncta were determined (*n* = 3; vehicle, 0.04 % DMSO; * *p* < 0.05, one-way ANOVA with post-hoc Newman–Keuls). **j**, **k** Primary neuroglial cultures were exposed to the indicated drug combinations (AZT, 25 μm; Rit, 10 μm; Saq, 1 μm) for 48 h, followed by hand counting for MAP2-positive cells (**j**) or MAP2 cell-based ELISA (**k**) (*n* = 2; vehicle,: 0.04 % DMSO; * *p* < 0.05; # *p* < 0.01, one-way ANOVA with post-hoc Newman–Keuls). **l** Primary neuroglial cultures that were exposed to the indicated drug combinations (AZT, 25 μm; Rit, 10 μm; Saq, 1 μm) for 16 h were analyzed for synaptophysin-positive puncta (*n* = 2; vehicle, 0.04 % DMSO; * *p* < 0.05, one-way ANOVA with post-hoc Newman–Keuls)

**Fig. 3:**
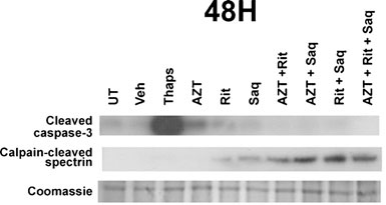
Activation of Calpain in antiretroviral drug-treated neurons. Whole cell lysates were prepared from neuroglial cultures treated with the indicated single or combination drugs (AZT, 25 μm; Rit, 10 μm; Saq, 1 μm), or with Thapsigargin (1 μM) as a positive control, for 48 h. Calpain activation was assessed using an antibody to detect the accumulation of calpain-cleaved spectrin and an antibody raised against the cleaved and active form of caspase-3 was used for detection of caspase activity. A band revealed by coomassie staining of the gel was used as a loading control (*n* = 2; vehicle, 0.04 % DMSO)

The only primary neural cell-type previously studied for ARV toxicity was dorsal root ganglia neurons in models of peripheral neuropathy (Werth et al. 1994). Here, to expand our studies on ARV neurotoxicity in the CNS, we used primary rat cortical neuroglial cultures aged 21 DIV (O'Donnell et al. 2006; Wang et al. 2007; White et al. 2011). We first evaluated the neuronal viability in response to increasing concentrations of AZT, Rit, or Saq. We based the range of doses on reported plasma and CSF levels of ARVs (Wynn et al. 2002; Anthonypillai et al. 2004). Importantly, animal studies predict brain parenchymal levels to be equal to or greater than CSF levels (Anthonypillai et al. 2004; Anderson and Rower 2010). At 48 h posttreatment with individual ARVs, Rit and Saq both led to dose-dependent decreases in MAP2-positive cells, as determined via hand counting (Fig. 2a, b, respectively). These results were confirmed with a cell-based MAP2 ELISA, which accurately reflects neuronal numbers, as well as neuronal damage (Fig. 2d, e) (White et al. 2011). Furthermore, neuronal damage and death induced by Rit and Saq was time dependent, as seen in Fig. 2g, where the primary cortical cultures were exposed to individual ARVs for up to 8 days. Additionally, both Rit and Saq led to dose-dependent decreases in synaptophysin expression after 16 h of treatment, well before the loss of MAP2-positive cells occurred (Fig. 2h, i). However, AZT did not lead to decreases in MAP2-positive cells, MAP2 fluorescence, or synaptophysin expression (Fig. 2c, f, and data not shown, respectively). Next, we treated primary cortical cultures with drug combinations that included AZT, Rit, and Saq, alone or in combinations, for 48 h, to assess acute neurotoxicity. As seen in Fig. 2j, k, combination treatments that included Rit induced statistically significant neuronal damage/death, which were preceded by loss of synaptophysin detected at 16 h posttreatment (Fig. 2l). Additionally, we observed similar levels of neuronal damage in cortical cultures treated with a combination of d4T, Rit, and Saq (not shown). However, as d4T is no longer prescribed in most developed countries and the AZT/Rit/Saq combination reflects the first considered/prescribed combination in initial treatment plans, we focused on AZT/Rit/Saq combination treatments for further experiments. Next, we determined that treatment with either Rit or Saq, alone or in combination with AZT, lead to the activation of the Ca^2+^-activated death protease, calpain, as evidenced by the increase of calpain-cleaved spectrin observed in these cultures (Fig. 3). Interestingly, none of the treatment combinations caused increases in the cleaved, and therefore active, form of caspase-3 (Fig. 3), suggesting that neuronal death observed in our model may be a necrotic cell death rather than an apoptotic cell death. Together the findings presented here suggest that combination ARV treatments that include PIs are toxic to cortical neurons in vitro.

### Combination antiretroviral drug treatments induce oxidative stress in neurons

**Fig. 4:**
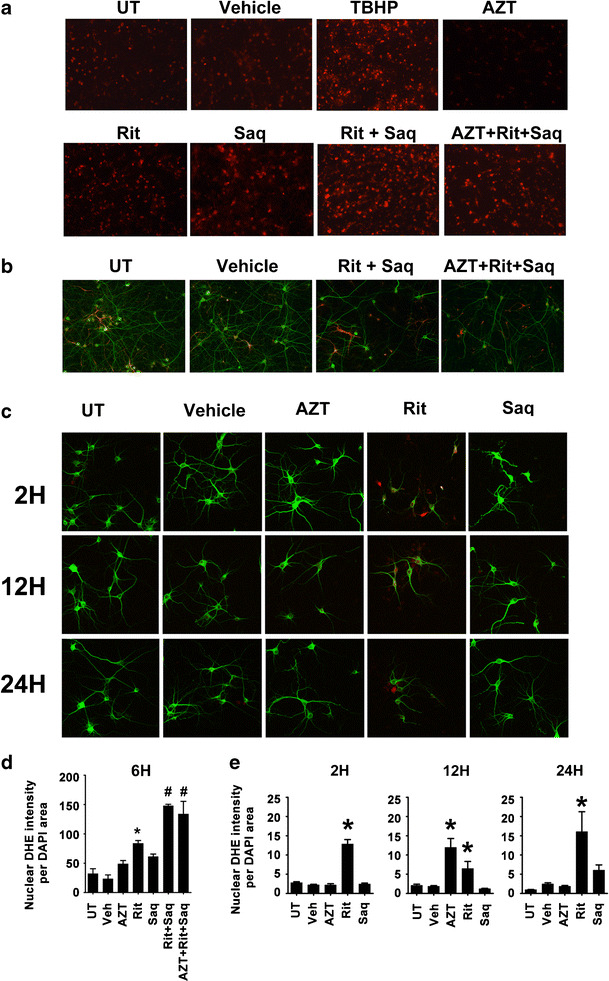
Combination antiretroviral drug treatments induce oxidative stress in neurons. **a**, **d** Cortical neuroglial cultures grown on coverslips were treated for 6 h with the indicated drugs (AZT, 25 μm; Rit, 10 μm; Saq, 1 μm) and the presence of ROS was detected by DHE staining (*red* fluorescence). **a** The images were captured with epifluorescent microscopy with uniform settings. **d** Quantification of DHE fluorescence was generated by measurement of DHE pixel intensity per DAPI area (*n* = 3; *p < 0.05; #*p* < 0.01, one-way ANOVA, post-hoc Newman–Keuls). **b** Cortical neuroglial cultures grown on coverslips for 21 days and exposed to the indicated treatments (AZT, 25 μm; Rit, 10 μm; Saq, 1 μm) were immunofluorescently labeled for MAP2 (*green*) and GFAP (*red*). Note that the cultures are enriched for neurons; **c** 21 DIV pure cortical neuronal cultures grown on coverslips were treated with AZT (25 μm), Rit (10 μm), or Saq (1 μm) for 2, 12, or 24 h. The presence of ROS was detected by DHE staining (*red* fluorescence) and MAP2 was used to stain neurons (*green* fluorescence). **e** The images captured with confocal microscopy with uniform settings were analyzed for DHE pixel intensity per DAPI area (*n* = 3; *p < 0.05, one-way ANOVA, post-hoc Newman–Keuls)

An extensive number of studies have linked ARVs, especially PIs, to oxidative stress (Touzet and Philips 2010). To determine whether neurons undergo oxidative stress when exposed to ARVs, we used dihydroethidium (DHE) as a marker for the presence of ROS (Rodriguez-Pallares et al. 2009). We included *tert*-butyl hydroperoxide, an organic pro-oxidant, at 100 μM, as a positive control. As seen in Fig. 4a and quantified in Fig. 4d, Rit/Saq and AZT/Rit/Saq combination drug treatments for 6 h lead to ROS accumulation in cortical cultures, whereas ROS levels in vehicle-treated cultures were comparable to those in untreated cultures.

**Fig. 5:**
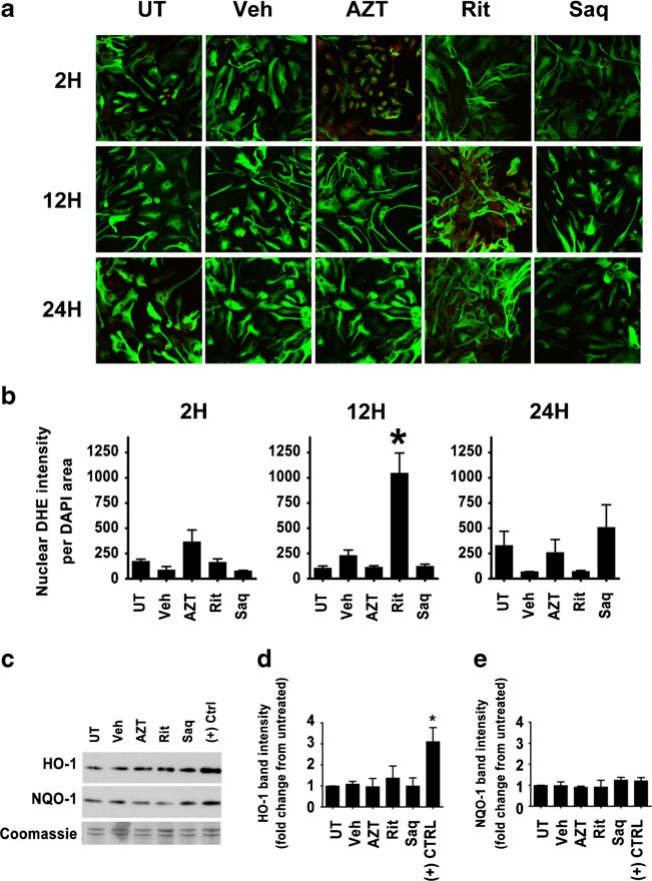
Antiretroviral drugs do not induce an endogenous antioxidant response in astrocytes. **a** Pure astrocytic cultures grown on coverslips were treated with AZT (25 μm), Rit (10 μm), or Saq (1 μm) for 2, 12, or 24 h. The accumulation of ROS was detected by DHE staining (*red* fluorescence) and GFAP was used to label astrocytes (*green* fluorescence). **b** The images captured with confocal microscopy with uniform settings were analyzed for DHE pixel intensity per DAPI area (*n* = 3; * *p* < 0.05, One-Way ANOVA, post-hoc Newman–Keuls). **c**–**e** Whole cell lysates from pure astrocytic cultures treated with the indicated drugs (AZT, 25 μm; Rit, 10 μm; Saq, 1 μm) for 16 h were immunoblotted for HO-1 and NQO-1. Representative blots from three independent experiments are shown in (**c**). Coomassie staining of gels were used as loading controls and fold changes over untreated lysates were determined. The quantification of HO-1 and NQO-1 band intensities from three independent experiments is shown in (**d**) and (**e**) (*n* = 3; *p < 0.05, one-way ANOVA, post-hoc Newman–Keuls)

The cortical cultures generated for our studies contain 85–90 % neurons and 10–15 % astrocytes (Fig. 4b); thus, we also determined the presence of ROS in pure neuronal and pure astrocytic cultures exposed to individual ARVs. As seen in Fig. 4c and quantified in Fig. 4e, Rit led to an early and sustained ROS production in pure neuronal cultures, whereas Saq-induced ROS accumulation occurred 24 h after treatment. The transient ROS accumulation induced by AZT was resolved at 24 h posttreatment. Conversely, in pure astrocytic cultures, none of the three ARVs examined induced an appreciable sustained ROS generation, and only Rit caused a transitory ROS accumulation (Fig. 5a, b). These data further suggest that the ARV-induced ROS production observed in primary cortical neuroglial cultures are occurring in neuronal cell populations.

### Endogenous antioxidant response activation by combination antiretroviral drugs

**Fig. 6:**
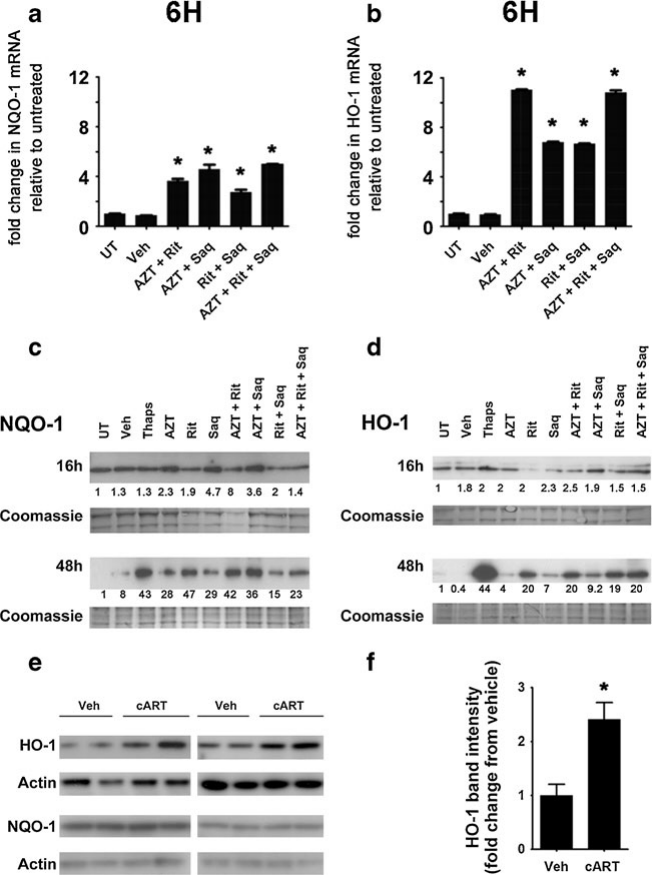
Combination antiretroviral drug treatments induce the endogenous antioxidant response in primary neuroglial cultures. **a**, **b** Cortical neuroglial cultures were exposed to the indicated treatments (AZT, 25 μm; Rit, 10 μm; Saq, 1 μm) for 6 h to determine changes in NQO-1 (**a**) and HO-1 (**b**) mRNA levels. A representative of three experiments is shown. Actin was used as internal control and fold changes were determined by the ΔΔC_T_ method (*p < 0.0001, one-way ANOVA, post-hoc Newman–Keuls). **c**, **d** Whole cell lysates from cortical neuroglial cultures treated with the indicated drug combinations (AZT, 25 μm; Rit, 10 μm; Saq, 1 μm) for 16 and 48 h were immunoblotted for NQO-1 (**c**) and HO-1 (**d**). Representative blots from three independent experiments are shown. Coomassie staining of gels were used as loading controls and fold changes over untreated lysates are indicated below each band of interest. **e**, **f** Whole cell lysates prepared from hippocampus of rats treated for 7 days with AZT/Rit/Saq (*n* = 4) or vehicle (*n* = 2) were immunoblotted for HO-1 and NQO-1 (**e**) and fold change in HO-1 protein levels in the cART group compared with the vehicle group is shown (**f**) (*p < 0.05, Student's *t* test)

Oxidative stress in cells triggers the transcriptional induction of oxidative stress responsive genes through the activation of the endogenous antioxidant response element in their promoter regions (Chen and Kong 2004). Thus, we determined the effect of ARVs on two such genes, NQO-1 and HO-1. By qRT-PCR, we observed increased mRNA levels of both NQO-1 and HO-1 in cultures treated with any of the tested combinations of ARVs, when compared with the levels in untreated cultures (Fig. 6a, b, respectively), with the most striking increases observed in Rit/Saq-treated cultures. To determine whether the changes in the mRNA levels were reflected in protein levels, we used immunoblotting to examine protein levels of NQO-1 and HO-1. While we observed increases in NQO-1 and HO-1 protein levels at 16 h (Fig. 6c), the changes were more robust in cultures treated for 48 h (Fig. 6d). Further, in vivo, we detected increased levels of HO-1 protein in hippocampal lysates from cART-treated rats (Fig. 6e, f). Interestingly, NQO-1 levels were not increased in cART-treated rat hippocampus (Fig. 6e). Of note, we did not observe an antioxidant response in ARV-treated pure astrocytic cultures (Fig. 5c–e). These data collectively suggest an activation of HO-1, as part of the endogenous antioxidant response, following ARV treatments in neurons.

### Monomethyl fumarate protects against ROS generation and induces activation of the endogenous antioxidant response

**Fig. 7:**
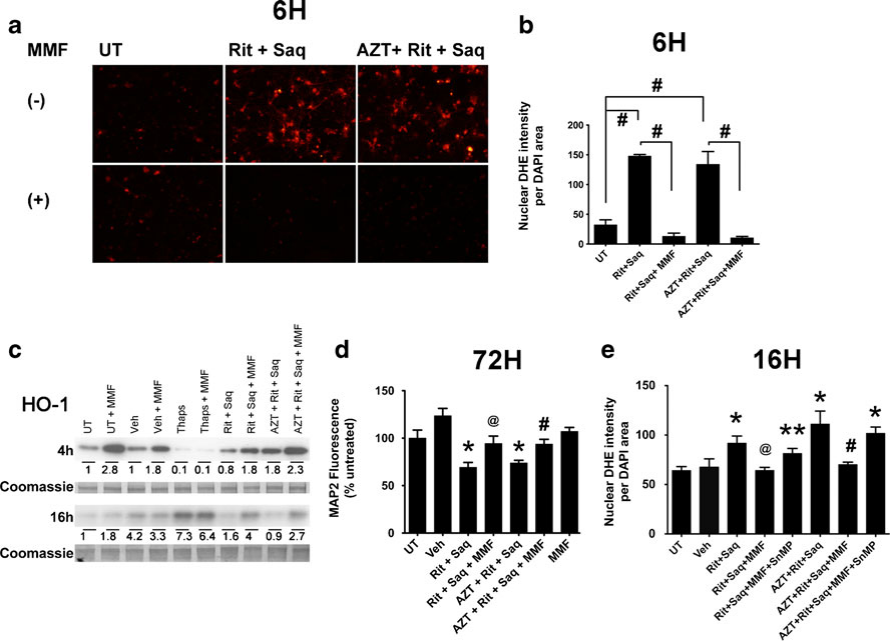
MMF induces activation of a cellular antioxidant response and blocks neuronal damage/death. **a** In the absence (*top*) or presence (*bottom*) of MMF (100 μM), 21 DIV rat neuroglial cultures on coverslips were treated for 6 h with Rit/Saq or AZT/Rit/Saq (AZT, 25 μm; Rit, 10 μm; Saq, 1 μm) or were left untreated. ROS generation was detected by DHE staining (*red* fluorescence). **b** Quantification of nuclear DHE was done as described above (*n* = 3; *p < 0.01, one-way ANOVA with post-hoc Newman–Keuls). **c** Whole cell lysates of cultures exposed to Rit/Saq or AZT/Rit/Saq treatments (AZT, 25 μm; Rit, 10 μm; Saq, 1 μm) in the absence or presence of MMF (100 μM) for 4 or 16 h were immunoblotted for HO-1. A representative blot from three independent experiments is shown. A coomassie band from the gel was used as loading control. Quantification of band intensities is shown under each corresponding lane. **d** Primary cortical neuroglial cultures were either pretreated with MMF (100 μM) for 30 min or received no pretreatment; cultures were then treated with Rit/Saq or AZT/Rit/Saq (AZT, 25 μm; Rit, 10 μm; Saq, 1 μm) for 72 h to assess neuronal damage/death by MAP2 ELISA (*n* = 3; vehicle = 0.04 % DMSO; *p < 0.05 vs. untreated; @*p* < 0.05 vs. Rit/Saq; #*p* < 0.05 vs. AZT/Rit/Saq, one-way ANOVA with post-hoc Newman–Keuls). **e** Primary cortical neuroglial cultures were preincubated with SnMP (20 μM) and/or MMF (100 μM) for 30 min before the addition of the indicated antiretroviral drugs (AZT, 25 μm; Rit, 10 μm; Saq, 1 μm) and were assessed for nuclear DHE accumulation at 16 (*n* = 3; *p < 0.01 vs. untreated; **p < 0.05 vs. untreated; @*p* < 0.01 vs. Rit/Saq; #*p* < 0.05 vs. AZT/Rit/Saq, one-way ANOVA with post-hoc Newman–Keuls)

NQO-1 and HO-1 are targets of the fumaric acid ester, dimethyl fumarate (DMF), and of its hydrolyzed and active metabolite, MMF (Linker et al. 2011). Our recent study (Cross et al. 2011) has shown that DMF and MMF reduce neurotoxin release from HIV-infected macrophages through induction of HO-1. We reasoned that augmented or earlier induction of NQO-1 and HO-1 in neurons by pretreatment with the active metabolite, MMF, would provide protection in our in vitro model of ARV-induced neurotoxicity through an antioxidant effect. We first determined that MMF at 30–100 μM was not toxic in neuronal cultures, as determined by MAP2 ELISA (data not shown). Next, we preincubated primary cortical cultures with 100 μM MMF for 30 min before adding either Rit/Saq or AZT/Rit/Saq. Preincubation with MMF blocked ROS generation induced by ARV treatments lasting 6 (Fig. 7a), 16, and 48 h (data not shown). Quantification showed ROS levels in cultures preincubated with MMF to be comparable to those detected in untreated and vehicle-treated cultures (Fig. 7b). Furthermore, we observed an early increase in HO-1 protein levels at 4 h in cultures preincubated with MMF, whether or not pretreatment was followed by ARV treatment (Fig. 7c). This trend was sustained at 16 h after combination ARV treatment, as well. Next, we determined whether augmenting the endogenous antioxidant response by MMF blocks MAP2 loss induced by combination ARV treatments. As detected by the cell-based MAP2 ELISA, MMF blocked neuronal damage/death in cultures treated with Rit/Saq and AZT/Rit/Saq for 72 h (Fig. 7d). Finally, pretreatment of the cultures with an HO-1 inhibitor, SnMP (Zhao et al. 2004), reversed the MMF-induced decreases in ROS accumulation (Fig. 7e), suggesting that MMF protection occurs through its augmentation of HO-1 expression.

## Discussion

Virus-related factors, such as resistant virus species and persistent viral DNA in the CNS, may contribute to the persistence of HAND in the post-cART era. One recent focus in HIV neurovirology is the development of ARVs with greater CNS penetrance (Letendre et al. 2008; Marra et al. 2009; Tozzi et al. 2009; Edén et al. 2010; Heaton et al. 2010; Garvey et al. 2011; Smurzynski et al. 2011). However, potential ARV toxicity in the CNS remains largely unexplored. Our study supports a possible contribution of the ARVs themselves to neuronal and synaptic damage observed in patients with HAND.

In this study, we show cART-induced synaptophysin loss, indicative of synaptic injury in two animal models. In our first model, in an in vivo model of SIV-infected pig-tailed macaques, we report decreased synaptophysin and CaMKII levels in the SIV(+)/cART group compared with uninfected or SIV(+)/placebo groups, indicating synaptodendritic damage. Interestingly, MAP2 levels did not change significantly across groups, which may be because of the relatively short duration of infection in this retrospective study cohort. While these data demonstrate potential effects of cART drugs in the presence of viral infection, there are three variables to consider in interpretation of these findings: (1) the time to euthanasia from the start of the experiments is different between SIV(+)/placebo and SIV(+)/cART groups, (2) persistent viral DNA in the CNS of SIV(+)/cART group, and (3) the lack of SIV(-)/cART group. It is not yet known whether brain SIV DNA is replication competent. As we utilized post-mortem samples obtained from a cohort of macaques enrolled in a previous study addressing the efficacy of CNS penetrant cART in reducing viral loads in the CNS, further experiments that include an additional control group receiving cART but not inoculation with SIV will be instrumental to more clearly determine the contribution of viral DNA and cART to synaptic damage in this model.

In the second in vivo model presented here, adult rats received a therapeutically relevant combination of ARVs (NRTI+PI+Rit boost). In the small number of studies where pharmacokinetics and effects of ARVs in the CNS were examined, pathological read-outs of neuronal damage, such as MAP2 or synaptophysin loss, were not determined (Huisman et al. 2003; Anthonypillai et al. 2004; Anthonypillai et al. 2006). Synaptic injury is a known indicator of neuronal damage and dysfunction in various neurodegenerative diseases, including HAND (Gupta et al. 2010) and synaptodendritic injury persists in HIV-infected individuals in the post-cART era (Xu and Ikezu 2009). We observed decreases in synaptophysin and MAP2 protein levels in the hippocampus in response to ARV administration over 7 days. Thus, our model demonstrates that ARV-associated neurotoxicity warrants consideration in developing therapeutic regimens for HIV-infected patients.

We also show that the PIs, Rit and Saq, alone or in combinations with the NRTI, AZT, induce oxidative stress, and neuronal damage/death in primary cultures at clinically relevant doses. Previous studies examined ARV-induced toxicity in cell lines, and Robertson et al. have provided the first evidence for ARV-induced neurotoxicity in primary rat neurons (Robertson et al. 2012). Here, we provide further evidence that PI-induced oxidative stress and neuronal death in primary neurons can be blocked by the activation of the endogenous antioxidant response. Interestingly, in our experimental paradigm, the NRTI, AZT, neither induced neuronal damage/death by itself, nor augmented PI-induced damage/death when used in combination. We observed similar effects from a combination using another NRTI, stavudine (d4T; not shown). In agreement with our observations, a previous study presented similar findings, specifically that neither AZT nor d4T inhibited cell growth or neurite regeneration in PC-12 cells after long-term drug exposure (Cui et al. 1997). It should be noted that NRTIs are unequivocally tied to peripheral neuropathy, where the underlying pathology is mitochondrial toxicity and oxidative stress. However, NRTIs affect only certain cell populations, as they are formulated as pro-drugs and, to become active, need to be phosphorylated by two kinases, thymidine kinase 1 and 2 (TK1 and TK2), and the expression of the cytoplasmic TK1 is cell cycle dependent (Bazzoli et al. 2010). Thus, in our model utilized to study post-mitotic neurons, AZT is most likely not converted to its active form, and thus does not contribute to neuronal damage/death. It is of note that our in vitro model of ARV-induced neurotoxicity utilized primary neuroglial cultures rather than cell lines. Primary cells are untransformed, and therefore more accurately reflect and predict ARV-associated effects occurring in the brains of patients on cART than would immortalized cell lines. The molecular pathways we investigated in this study are highly conserved from yeast to human cells; thus, the results obtained here in cells of rodent origin are likely conserved in human cells as well.

The drug concentrations used in this study are based on the plasma and CSF levels reported by various in vitro and in vivo studies(Huisman et al. 2003). As reported in such studies, AZT can be detected in the CSF at concentrations that are similar to those measured in the plasma (Wynn et al. 2002). Contrarily, as backed by various in vitro and in vivo studies (Wynn et al. 2002), both Rit and Saq are predicted to have limited CNS penetrance due to the strong tendency of these drugs to bind plasma protein because of their lipophilic nature and pharmacokinetic properties. However, a comprehensive study conducted in an in situ guinea pig model suggests that Rit can achieve high concentrations in choroid plexus and parenchyma through diffusion via the choroid plexus; in fact, the levels of Rit were comparable to levels measured in plasma (Anthonypillai et al. 2004). Furthermore, the study showed that, surprisingly, the CSF levels of Rit were lower than the levels measured in the choroid plexus and the parenchymal compartments. Thus, the authors concluded that CSF concentrations of Rit may not necessarily reflect the parenchymal levels, which are indeed a better indicator of effective drug levels in the CNS. Rit and Saq concentrations in our experiments fall well within plasma ranges reported in patients receiving cART.

Evidence of oxidative stress has long been associated with the most severe forms of HAND (Ngondi et al. 2006; Mielke et al. 2010). Interestingly, despite systemic control of viral replication in cART-treated patients, markers of oxidative stress, such as elevated levels of lipid and protein oxidation, are still detectable in the brains of these individuals (Ances et al. 2008). Of note, oxidative stress is one of the underlying mechanisms involved in NRTI and PI-induced toxicity in the periphery. Our data suggest that the sustained ROS accumulation in neurons due to prolonged exposure to ARVs might induce the oxidative stress associated with cART-induced toxicity in the CNS, leading to the observed changes in synaptophysin levels and the subsequent neuronal death.

Our data also suggest that astrocytes do not show neither ROS accumulation nor the endogenous antioxidant response in vitro. We also observe that astrogliosis in SIV-infected animals is resolved in cART-treated animals. While these findings suggest that astrocytes may not be highly impacted by ARVs in the short term, it is possible that prolonged exposure to ARVs might overwhelm astrocytes, which help buffer ROS accumulation in neurons under normal conditions, precipitating further neuronal damage.

We further show that the ARV drug-induced effects observed in this study can be blocked by MMF via activation of an endogenous antioxidant response. Cells respond to oxidative stress by activating the transcription of a subset of genes in an effort to clear excess ROS within the cell. Among these genes are NQO-1 and HO-1 (Chen and Kong 2004). Here, we show increased levels of NQO-1 and HO-1 mRNA and protein in ARV-treated neuronal cultures. Additionally, pretreatment of cultures with MMF led to further increases in HO-1 protein levels in ARV-treated cultures and provided protection against ARV-induced toxicity. Finally, our finding that the protection provided by MMF is reversed with chemical inhibition of HO-1 further provides evidence that ARV toxicity is mediated via induction of oxidative stress. The neuronal damage and death that occurs despite the cellular initiation of the endogenous antioxidant response following exposure to ARVs suggests that this response may be insufficient or too delayed to protect cultures from ARV toxicity. In support of this explanation, we observed that MMF-mediated augmentation of antioxidant responses is strongly protective against the neurotoxic effects of ARVs. Further studies using this in vitro model are warranted to determine whether other clinically relevant ARV drug combinations induce neurotoxicity and, if so, to establish whether similar pathways are involved.

DMF, a psoriasis treatment used in Europe since 1994, is currently being tested as a disease-modifying agent for multiple sclerosis (MS) (Anonymous 2011; Krieger 2011; Linker et al. 2011). MMF is the active DMF metabolite in vivo. Our report is the first to show MMF as a neuroprotectant against ARV-induced damage. Data from studies in MS patients show that DMF/MMF has good tolerability, can cross the BBB efficiently, and has relatively few and minor side effects. Furthermore, we have recently shown that DMF suppresses HIV replication, induces the antioxidant response in macrophaghes, and blocks neurotoxin release from macrophages (Cross et al. 2011). Overall, our findings make this immunomodulatory and antioxidant agent a good potential adjunctive therapeutic for use in HAND.

One of the paradoxical outcomes of cART is the persistence of HAND, despite successful viral control. Moreover, there is a shift from overt dementia to subtler neurocognitive impairments. Based on data presented here, we propose that different mechanisms by which HIV infection and cART induce neuronal damage underlie the changing neuropathology of HAND. Extensive studies have shown that during lentiviral infection, viral proteins and soluble factors secreted by infected cells lead to neuronal and synaptic damage via several direct as well as indirect mechanisms involving several cell types, including astrocytes. However, our data suggest that cART-mediated synaptic damage may involve direct mechanisms occurring specifically in neurons, such as oxidative stress, and that neurons, and not astrocytes, are the primary targets of cART-mediated damage in the CNS.

It is most likely that chronic exposure to cART regimens including neurotoxic ARVs over many years is associated with a slower, nonetheless insidious changes including synaptic damage in the absence of neuronal loss, and these neuronal perturbations may contribute to, and may even precipitate, some of the clinical and pathological changes observed in the chronic course of HAND in the cART era. While detrimental, such slow damage also suggests that alterations in cART regimens to include ARVs with low neurotoxicity profiles, such as NRTIs or nNRTIs may halt synaptic damage, and provide a point where previous damage may be reversed, either due to the withdrawal of the toxic drug, or with the help of an adjuvant, such as fumaric acid esters. Future studies, first in primates, then in humans, will be crucial to explore the specific impact of treatment interruption on recovery from cART-mediated neuronal damage and to determine the eddicacy of potential adjunctive therapies necessary to mitigate the side effects of cART in the CNS.
